# Supplementary material for: Another dengue fever outbreak in Eastern Ethiopia—An emerging public health threat
Source: PLoS Negl Trop Dis. 2021 Jan 19;15(1):e0008992. doi: 10.1371/journal.pntd.0008992 (PMC7845954; doi:10.1371/journal.pntd.0008992)
Supplement: S1 Table — (DOCX) [file pntd.0008992.s001.docx]

**S1 Table:** Entomological Survey - Kabridahar District, Korahay Zone, Somali Region, Ethiopia, 2017

| **Sampled villages** | **Sampled houses** | **Houses with larvae** | **Containers with water** | **Containers with larva** | ***Aedes* (Stegomyia) Indices** | | | **Species identified** | | **Total** |
| --- | --- | --- | --- | --- | --- | --- | --- | --- | --- | --- |
|  |  |  |  |  | *House index*  *(HI)* | *Container index*  *(CI)* | *Breateu index*  *(BI)* | *Ae.*  *aegypti*  *No. (%)* | *Ae. africanus No. (%)* |  |
| 7 | 30 | 19 | 86 | 55 | 63 | 64 | 183 | 18 (86) | 3 (14) | 21 |
| 1 | 18 | 11 | 60 | 26 | 61 | 43 | 145 | 26 (93) | 2 (7.1) | 28 |
| 3 | 17 | 9 | 56 | 28 | 53 | 50 | 165 | 22 (96) | 1 (4.4) | 23 |
| 4 | 15 | 7 | 46 | 22 | 46 | 48 | 147 | 12 (75) | 4 (25) | 16 |
| 2 | 18 | 8 | 62 | 30 | 44 | 48 | 167 | 16 (94 | 1 (5.9) | 17 |
| 9 | 5 | 2 | 15 | 6 | 40 | 40 | 120 | 14 (88) | 2 (13) | 16 |
| 10 | 6 | 2 | 20 | 8 | 33 | 40 | 133 | 12 (100) | 0 | 12 |
| 6 | 12 | 4 | 27 | 14 | 33 | 51 | 117 | 10 (100) | 0 | 10 |
| 5 | 10 | 3 | 28 | 16 | 30 | 57 | 160 | 9 (90) | 1 (10) | 10 |
| 8 | 5 | 1 | 11 | 5 | 20 | 45 | 100 | 12 (86) | 2 (14) | 14 |
| **Total** | **136** | **66** | **411** | **210** | **49** | **51** | **154** | **151 (90)** | **16 (9.6)** | **167** |
