# Supplementary material for: Another dengue fever outbreak in Eastern Ethiopia—An emerging public health threat
Source: PLoS Negl Trop Dis. 2021 Jan 19;15(1):e0008992. doi: 10.1371/journal.pntd.0008992 (PMC7845954; doi:10.1371/journal.pntd.0008992)
Supplement: Attachment — Submitted filename: PNTD-D-20-00543_Comments_MD.pdf [file pntd.0008992.s002.pdf]

The manuscript entitled "Another Dengue Fever Outbreak in Eastern Ethiopia – An Emerging Public Health Threat" described an outbreak investigation and characterization eastern Ethiopia in May 2017. Indeed, in Africa, there are no formal dengue surveillance systems which, together with lack of laboratory diagnostic capacities in most countries lead to the underestimation of dengue burden. Therefore, and despite the limitations of this study, it is an important addition to the literature of dengue in Ethiopia and in Africa as a whole. However, I have to suggest that the overall scientific quality be improved.

Major points

1. The authors should explain laboratory investigations by providing details on the CDC trioplex method, together with the serotyping techniques.
2. Authors are strongly advice to do sequencing of the circulating dengue strain as it is important for tracking the movement and evolution of these viruses.
3. The status of dengue in Ethiopia is not well documented in the manuscript despite several and recently published articles on dengue in Ethiopia: two from Ferede et al. (2018), Degife et al. (2019, Mohammed Yusuf et al. (2019), Mengesha Tsegaye et al. (2018), Geleta, (2019), etc.
4. There is no information on the index case. This could help better understand the outbreak.
5. Details should be given on the 5 severe cases with emphasis on the death patient. For instance, were they all PCR positive, was any autopsy done on the corps to identify the cause of the death? Etc.
6. The authors should consider restructuring their discussion in a chronological order according the results section and, also revising the discussion which is focused mainly on the vector aspects.

Minor points

Line numbers are not included in the manuscript file making review a bit difficult. Also, there are a few grammatical mistakes throughout the manuscript.

ABSTRACT

A total of 101 total cases… Please remove one total.

AUTHOR SUMMARY

- Please rephrase this: We implemented measures to combat or promote these issues and after five days cases reduced.
- This mosquito transmitted disease was recently introduced in Ethiopia only four years prior, with this being the first time it was identified in the area.

Is there any reference supporting this introduction? Was the disease recently introduced or detected?

INTRODUCTION

- Repeat infections… Please write repeated infections
- DENV is endemic… The disease is endemic not the virus!
- This is particularly important, as the weather conditions in the region result in the common practice of early morning and daytime sleeping, which coincides with the feeding and activity patterns of the *Ae. aegypti* vector.

Please provide reference for this. Which region is concern?

METHODS

- Where is situated the epicenter of the previous outbreak compared to the one described in this manuscript? This should appear on Ethiopia map on Figure 2.
- Laboratory investigation: provide details on CDC trioplex, see example of Sado et al (2018).
- Give a brief description of the serotyping method used
- Among the collected mosquitoes, *Aedes* mosquitoes belonging to two species, namely *Ae. aegypti* and *Ae. africanus*, were identified using a standard morphological identification key developed by Rueda, 2004

If the Rueda's key was used only for Ae aegypti and africanus how were Aedes identified among all the mosquitoes collected?

- Ethical approval: provide reference of the letter from Health authorities.

RESULTS

- Nothing is said about rainfall and cases as presented in Figure 1
- No detail on index case?
- Five cases presented warning signs, demonstrating epistaxis or gingival bleeding. One case presented with thrombocytopenia. Of these, three were referred to Jijiga Regional Referral Hospital and one (41-year-old male) died.

Was the death patient the one presenting with thrombocytopenia?

- It is not clear whether the 21 samples include the 5 severe cases or not and if the dead patient was confirmed dengue, and if any autopsy was done on this case.
- Ferede et al. (2018) found high risk of dengue in August (summer with heavy rain falls) and May (Spring and hottest month). Could you please use this article to support your results?
- How do you explain the presence of Ae africanus in town?

DISCUSSION

- Your conclusions cannot affect the existing knowledge in the field if you don't consider other articles previously or recently published in this subject in Ethiopia. Moreover, your research must be built on previous observations.
- It is not clear whether the previous dengue outbreak was in 2013 or 2014!
- The low severe dengue rate in this outbreak along with the corresponding low case-fatality rate suggests a new introduction of DV to this area, and few, if any prior, infections among the population.

This cannot be a new introduction of DENV in the area since you wrote (end of page 11) that Ahmed et al. (2016) revealed DENV circulation in Somali region in 2014!

Serological tests would have been helpful in the understanding of this hypothesis.

- Consider making one sentence with these two: It is common for the residents. Of this community to participate in early morning and day sleeping to avoid the harsh weather associated with this region.

REFERENCES

- Follow PLOS NTD recommendations for online references. How can a reference be accessed 3 years before outbreak onset?
