# Supplementary material for: Another dengue fever outbreak in Eastern Ethiopia—An emerging public health threat
Source: PLoS Negl Trop Dis. 2021 Jan 19;15(1):e0008992. doi: 10.1371/journal.pntd.0008992 (PMC7845954; doi:10.1371/journal.pntd.0008992)
Supplement: Attachment — Submitted filename: Response to Reviewers.docx [file pntd.0008992.s003.docx]

**Response to Reviewer’s Comments**

PNTD-D-20-00543

Another Dengue Fever Outbreak in Eastern Ethiopia – An Emerging Public Health Threat”

A detailed response to each comment is listed below. All changes in response to the reviewer’s comments have been incorporated into the manuscript text.

**Major points**

**1. The authors should explain laboratory investigations by providing details on the CDC trioplex method, together with the serotyping techniques.**

Thank you for the suggestion. This has been addressed in lines 189-216 of the revised manuscript.

**2. Authors are strongly advice to do sequencing of the circulating dengue strain as it is important for tracking the movement and evolution of these viruses.**

We would like to thank the reviewer for this comment. We agree with the reviewer’s suggestion regarding the importance of sequencing positive samples. We regret to reveal however, that this is not possible due to resource limitations in the country. As of now, there are no sequencing facilities or capacity at our disposal within Ethiopia. This is admittedly a limitation of our study and makes portions of our laboratory results observational and correlative when it comes to their relationship to past Dengue outbreaks. However, given the similar serotype makeup within the country and Somali Region, as identified by Ahmed et al. 2016, we feel that it is important to share our significant, albeit serologically-limited, findings. The timing, location, and risk factors associated with this outbreak add valuable information to the slowly growing database of Dengue in Ethiopia and can provide valuable insight toward future surveillance and prevention efforts. All positive samples are being stored in -80^o^C with the plan to undergo sequencing when such capacities are in place. A future update may be possible as a result. This point has been added to the discussion section of the revised manuscript in lines 459-460.

**3. The status of dengue in Ethiopia is not well documented in the manuscript despite several and recently published articles on dengue in Ethiopia: two from Ferede et al. (2018), Degife et al. (2019, Mohammed Yusuf et al. (2019), Mengesha Tsegaye et al. (2018), Geleta, (2019), etc.**

We thank the reviewer for the suggestion. A more complete picture of the Dengue fever outbreak history within Ethiopia has been added in lines 115-127 of the revised manuscript. Additionally, a thorough review of relevant literature on the subject resulted in multiple additions and expansions to the discussion section in the revised manuscript. The following subjects and corresponding line locations in the revised manuscript have been added:

- Under reporting of infections- 347-351
- Serological information- 351-357
- Influence of education- 377-384
- Influence of sex- 389-393
- Influence of religious practices-393-398
- Larval density indices- 408-418
- Influence of rainfall and timing- 425-432
- Influence of LLITNs- 433-445

**4. There is no information on the index case. This could help better understand the outbreak.**

Thank you for comment. The reviewer is correct that information on the index case could be a beneficial component for better understanding the outbreak. This has been addressed in lines 288-291 of the revised manuscript.

**5. Details should be given on the 5 severe cases with emphasis on the death patient. Ex: were they all PCR positive, was any autopsy done on the corps to identify the cause of the death, etc?**

Thank you for comment. The reviewer is correct that further information on the severe cases could be beneficial information. This has been addressed in lines 299-309 of the revised manuscript.

**6. The authors should consider restructuring their discussion in a chronological order according the results section and, also revising the discussion which is focused mainly on the vector aspects.**

This is an excellent point, and we thank you for the comment. The discussion in the revised manuscript has been adjusted to reflect the order described in the results section and to incorporate other aspects of the investigation; lines 340-489.

**Minor points**

**Line numbers are not included in the manuscript file making review a bit difficult. Also, there are a few grammatical mistakes throughout the manuscript.**

Thank you for catching this oversight. This has been corrected in the revised manuscript.

**ABSTRACT**

**A total of 101 total cases… Please remove one total.**

We appreciate the reviewer’s comment, yet respectfully disagree with their sentiments. We decided to utilize the entirety of our study sample and to not remove 1 case. We agree 100 is a much more navigable and simplistic sample size to quickly understand, but felt the data is better served by including the entire sample.

**AUTHOR SUMMARY**

**Please rephrase this: We implemented measures to combat or promote these issues and after five days cases reduced.**

Thank you for the suggestion. This has been corrected in the revised manuscript in lines 85-87.

**This mosquito transmitted disease was recently introduced in Ethiopia only four years prior, with this being the first time, it was identified in the area. Is there any reference supporting this introduction? Was the disease recently introduced or detected?**

This is an excellent point, and we appreciate the comment. We do not have any concrete evidence that the infection was introduced, thus this cannot be proven in our study. The wording has been amended to reflect first detection in lines 75 and 115 of the amended manuscript. References to the initial identification of the infection can be seen in the reference section: References 15 (WHO 2014) and 18 (Ahmed et al. 2016).

**INTRODUCTION**

**Repeat infections… Please write repeated infections**

Thank you for catching this oversight. This has been corrected in line 98 of the revised manuscript.

**DENV is endemic… The disease is endemic not the virus!**

Thank you for catching this oversight. This has been corrected in line 101 of the revised manuscript.

**This is particularly important, as the weather conditions in the region result in the common practice of early morning and daytime sleeping, which coincides with the feeding and activity patterns of the *Ae. aegypti* vector. -Please provide reference for this. Which region is concern?**

Thank you for the suggestion. The wording has been amended to specify the Somali Region in lines 111-113 of the revised manuscript. A reference does not currently exist for this anthropological practice and was instead observed by the field team during the outbreak investigation. This important point has been added in lines 436-443 to the revised manuscript.

**METHODS**

**Where is situated the epicenter of the previous outbreak compared to the one described in this manuscript? This should appear on Ethiopia map on Figure 2.**

Thank you for comment. The reviewer is correct that the location of the previously discussed important outbreaks should be discussed. This information was added to the discussion portion of the revised manuscript in lines 342-344 and 462-464 where it best fits the narrative. Additionally, Figure 2 has been amended to display the location of key previous outbreaks in 2013 and 2014. This can be seen in Figure 2B of the amended figure document.

**Laboratory investigation: provide details on CDC trioplex, see example of Sado et al (2018).**

Thank you for the suggestion. This has been addressed in lines 189-205 of the revised manuscript.

**Give a brief description of the serotyping method used**

Thank you for the suggestion. This has been addressed in lines 206-216 of the revised manuscript.

**Among the collected mosquitoes, *Aedes* mosquitoes belonging to two species, namely *Ae.***

***aegypti* and *Ae. africanus,* were identified using a standard morphological identification key**

**developed by Rueda, 2004. If the Rueda’s key was used only for Ae aegypti and africanus** **how were Aedes identified among all the mosquitoes collected?**

Thank you for comment. Trained entomologists were able to morphologically identify specimens to the *Aedes* genus-level by sight using important morphological characteristics. This information is reflected in lines 241-244 of the revised manuscript.

**Ethical approval: provide reference of the letter from Health authorities.**

We would like to thank the reviewer for this comment This ethical approval was referenced in lines 269-275 of the revised manuscript, however this is not publicly available as it is in the possession of the Ethiopian health sector. We obtained a letter from The Ethiopian Public Health Institute, a legally authorized Institute to conduct outbreak investigations and to provide ethical clearance to conduct these investigations, as well as a letter from the Regional Health Bureau to obtain approval for data collection. Additional permission was also sought from Kabridahar District Health Office. This letter can be provided to the journal if necessary.

**RESULTS**

**Nothing is said about rainfall and cases as presented in Figure 1**

Thank you for the suggestion. The influence of rainfall on Dengue fever is now mentioned in lines 333-338 in the results section of the newly revised manuscript. Further information has also been included in lines 425-432 of the discussion portion of the newly revised manuscript.

**No detail on index case?**

Thank you for comment. The reviewer is correct that information on the index case could be a beneficial component for better understanding the outbreak. This has been addressed in lines 288-291 of the revised manuscript.

**Five cases presented warning signs, demonstrating epistaxis or gingival bleeding. One case**

**presented with thrombocytopenia. Of these, three were referred to Jijiga Regional Referral**

**Hospital and one (41-year-old male) died. Was the death patient the one presenting with thrombocytopenia?**

Thank you for comment. The reviewer is correct that further information on the severe cases could be beneficial information. This has been addressed in lines 299-309 of the revised manuscript

**It is not clear whether the 21 samples include the 5 severe cases or not and if the dead patient was confirmed dengue, and if any autopsy was done on this case.**

We appreciate the reviewer’s comment and have amended the manuscript in lines 299-309 to more clearly reflect the cases included and to convey the infective status of all cases, including that of the deceased patient.

**Ferede et al. (2018) found high risk of dengue in August (summer with heavy rain falls) and**

**May (Spring and hottest month). Could you please use this article to support your results?**

We would like to thank the reviewer for this comment. Information from this publication can now be found as a supporting reference in lines 335-338 of the revised manuscript.

**How do you explain the presence of Ae africanus in town?**

We appreciate the reviewer’s comment. Through our entomological surveillance it was discovered that *Aedes Africanus* was not present in Kabridahar town but instead in and around the homes located in the border areas of the town. These border areas are forested and more conducive to the presence of *Aedes Africanus*. This information was reflected in lines 248-250 of the revised manuscript.

**DISCUSSION**

**Your conclusions cannot affect the existing knowledge in the field if you dont consider other articles previously or recently published in this subject in Ethiopia. Moreover, your research must be built on previous observations.**

This is an excellent point and we thank the reviewers for the comment. A thorough review of relevant literature on the subject resulted in multiple additions and expansions to the discussion section in the revised manuscript. The following subjects and corresponding line locations have been added as result:

- Historical Dengue outbreaks in Ethiopia- 115-127
- Under reporting of infections- 347-351
- Serological information- 351-357
- Influence of education- 377-384
- Influence of sex- 389-393
- Influence of religious practices-393-398
- Larval density indices- 408-418
- Influence of rainfall and timing- 425-432
- Influence of LLITNs- 433-445

**It is not clear whether the previous dengue outbreak was in 2013 or 2014!**

Thank you for addressing this unclear description of historical outbreaks. We have clarified this issue in lines 117-121 and 342-343 of the revised manuscript. It should be clear now that outbreaks occurred in both 2013 and 2014, however these occurred in different locations.

**The low severe dengue rate in this outbreak along with the corresponding low case-fatality rate suggests a new introduction of DV to this area, and few, if any prior, infections among the population. This cannot be a new introduction of DENV in the area since you wrote (end of page 11) that Ahmed et al. (2016) revealed DENV circulation in Somali region in 2014!**

We appreciate the reviewer’s comment and have edited the content of this statement to be more specific. The content now reflects the first outbreak in Kabridahar Town on line 456 of the revised manuscript. It is true Dengue fever was recorded in the region previously, however this occurred in a more remote area to the southwest. This information, along with a possible route of introduction, was added to lines 462-465 of the revised manuscript.

**Serological tests would have been helpful in the understanding of this hypothesis.**

We appreciate the reviewer’s comment and agree with their sentiments. The lack of serological testing is a limitation of the study and is discussed as such in lines 459-460 of the revised manuscript.

**Consider making one sentence with these two: It is common for the residents. Of this**

**community to participate in early morning and day sleeping to avoid the harsh weather**

**associated with this region.**

This is an excellent point and has been corrected in lines 440-442 of the revised manuscript.

**REFERENCES**

**Follow PLOS NTD recommendations for online references. How can a reference be accessed 3 years before outbreak onset?**

We apologize for the oversight and have revised this, along with the entire references section (lines 560 – 69	1), to reflect PLOS NTD recommendations.

Reviewer's Responses to Questions

**Key Review Criteria Required for Acceptance?**

As you describe the new analyses required for acceptance, please consider the following:


**Methods**

-Are the objectives of the study clearly articulated with a clear testable hypothesis stated?

-Is the study design appropriate to address the stated objectives?

-Is the population clearly described and appropriate for the hypothesis being tested?

-Is the sample size sufficient to ensure adequate power to address the hypothesis being tested?

-Were correct statistical analysis used to support conclusions?

-Are there concerns about ethical or regulatory requirements being met?


**Reviewer #1: The author described, the RT-PCR Assay provided by CDC. Stating this is not relevant.**

Thank you for the suggestion. This has now been removed.

**Reviewer #2: The objectives of the study are clearly articulated and the study design is appropriate.
Correct statistical analysis were used to support conclusions
However, details of the laboratory methods are required to better understand the outbreak.**

Thank you for the kind words and beneficial suggestion. The laboratory methods have been addressed in lines 189-216 of the revised manuscript.

--------------------

**Results**

-Does the analysis presented match the analysis plan?

-Are the results clearly and completely presented?

-Are the figures (Tables, Images) of sufficient quality for clarity?

**Reviewer #1: Describe the relationship between rainfall and outbreak i.e. after how many weeks of rainfall end up/reduced did the outbreak started and the like.**

Thank you for the suggestion. The influence of rainfall on Dengue fever is now mentioned in lines 333-338 in the results section of the newly revised manuscript. Further information has also been included in lines 425-432 of the discussion section of the newly revised manuscript.

**The first sentence of the last paragraph said "In response to the epidemic, from May 15 – June 17, 2007". Is this year correct? ~287)**

Thank you for catching this oversight. This has been corrected in line 328 of the revised manuscript.

**Reviewer #2: The analysis presented matches the analysis plan and the results are clearly but not completely presented as details on the index case is missing as well as details on the severe cases and/or death case together with their laboratory results.**

Thank you for comment. The reviewer is correct that information on the index and severe cases could be a beneficial component for better understanding the outbreak. Information on the index case has been addressed in lines 288-291 of the revised manuscript. Further information on the severe cases are addressed in lines 299-309 of the revised manuscript.

**Figure 2 should highlight the location of previous outbreaks**.

Figure 2 has been amended to display the location of key previous outbreaks in 2013 and 2014. This can be seen in Figure 2B of the amended figure document.

--------------------

**Conclusions**

-Are the conclusions supported by the data presented?

-Are the limitations of analysis clearly described?

-Do the authors discuss how these data can be helpful to advance our understanding of the topic under study?

-Is public health relevance addressed?

**Reviewer #1: The recommendation of health care worker education is not supported by the findings in this study.**

We would like to thank the reviewer for this comment. A thorough review of relevant literature on the subject resulted in multiple additions and expansions to the education component of the investigation and can be found in lines 377-384 of discussion section in the revised manuscript.

**Reviewer #2: The conclusion are supported by the data presented and the limitations are clearly described. The public health relevance of the study has been addressed. However, discussion of these data compare to other available and published data in the country is missing.**

We thank the reviewer for the suggestion. A more complete picture of the Dengue fever outbreak history within Ethiopia has been added in lines 115-127 of the revised manuscript. Additionally, a thorough review of relevant literature on the subject resulted in multiple additions and expansions to the discussion section in the revised manuscript. The following subjects and corresponding line locations in the revised manuscript have been added as result:

- Under reporting of infections- 347-351
- Serological information- 351-357
- Influence of education- 377-384
- Influence of sex- 389-393
- Influence of religious practices-393-398
- Larval density indices- 408-418
- Influence of rainfall and timing- 425-432
- Influence of LLITNs- 433-445

--------------------

**Editorial and Data Presentation Modifications?**

Use this section for editorial suggestions as well as relatively minor modifications of existing data that would enhance clarity. If the only modifications needed are minor and/or editorial, you may wish to recommend “Minor Revision” or “Accept”.

**Reviewer #1: (No Response)**

**Reviewer #2: A few major revisions are needed**.

Thank you for the suggestion. All major revisions have been addressed in the Major points portion of this document.

**Summary and General Comments**

Use this section to provide overall comments, discuss strengths/weaknesses of the study, novelty, significance, general execution and scholarship. You may also include additional comments for the author, including concerns about dual publication, research ethics, or publication ethics. If requesting major revision, please articulate the new experiments that are needed.

**Reviewer #1: (No Response)**

**Reviewer #2: The study was overall well conducted although several major points need to be addressed. Authors are strongly advice to do sequencing of the circulating dengue strain as it is important for tracking the movement and evolution of these viruses.**

We would like to thank the reviewer for this comment, as well as for the kind words. We agree with the reviewer’s suggestion regarding the importance of sequencing positive samples. We regret to reveal however, that this is not possible due to resource limitations in the country. As of now, there are no sequencing facilities or capacity at our disposal within Ethiopia. This is admittedly a limitation of our study and makes portions of our laboratory results observational and correlative when it comes to their relationship to past Dengue outbreaks. However, given the similar serotype makeup within the country and Somali Region by Ahmed et al. 2016 we feel that it is important to share our significant, albeit serologically-limited, findings. The timing, location, and risk factors associated with this outbreak add valuable information to the slowly growing database of Dengue in Ethiopia and can provide valuable insight toward future surveillance and prevention efforts. All positive samples are being stored in -80^o^C with the plan to undergo sequencing when such capacities are in place. A future update may be possible as a result. This point has been added to the discussion section of the revised manuscript in lines 459-460.
--------------------
