# Supplementary material for: Another dengue fever outbreak in Eastern Ethiopia—An emerging public health threat
Source: PLoS Negl Trop Dis. 2021 Jan 19;15(1):e0008992. doi: 10.1371/journal.pntd.0008992 (PMC7845954; doi:10.1371/journal.pntd.0008992)
Supplement: Attachment — Submitted filename: PNTD-D-20-00543_R1_Comments_MD.docx [file pntd.0008992.s004.docx]

I have asked to revise the manuscript “Another Dengue Fever Outbreak in Eastern Ethiopia – An Emerging Public Health Threat”. I thank the authors for their responses and clarifications. The revised version of the manuscript is greatly improved. Although I still have some minor comments in order for the authors to fine-tune the manuscript, it can be considered suitable to be published in PNTD.

**Major points**

All my major comments were addressed except the one on sequencing, and although I understand the reason raised by the authors (no sequencing facilities or capacity in the country), I think this should not prevent a researcher from finding a solution, especially with many commercial companies available at affordable cost in Europe.

**Minor points (New)**

I kindly advise the authors to consider reading and taking into consideration a recently published systematic review on dengue prevalence in Africa by Simo Nemg et al. 2019 in their introduction or discussion.

Lines 150-152: I think this sentence sounds like a result, so please could you consider moving it to the appropriate section?

242-250: This paragraph of the method is a kind of results/discussion, so I kindly advise the authors to put it in the appropriate section.

Line 299: Please correct this sentence “Five cases of these cases presented…” as follow: “Five of the cases presented…”

**My Previous (minor) comment:**

Abstract: A total of 101 total cases… Please remove one total.

- Response of authors: We appreciate the reviewer’s comment, yet respectfully disagree with their sentiments. We decided to utilize the entirety of our study sample and to not remove 1 case. We agree 100 is a much more navigable and simplistic sample size to quickly understand, but felt the data is better served by including the entire sample.

My new comment:

The authors did not understand my comment here. I asked the authors to rephrase the sentence as follow: “A total of 101 cases…” without writing “total” twice.
