# Supplementary material for: Another dengue fever outbreak in Eastern Ethiopia—An emerging public health threat
Source: PLoS Negl Trop Dis. 2021 Jan 19;15(1):e0008992. doi: 10.1371/journal.pntd.0008992 (PMC7845954; doi:10.1371/journal.pntd.0008992)
Supplement: Attachment — Submitted filename: Response to Reviewers.docx [file pntd.0008992.s005.docx]

**Response to Reviewer’s Comments**

PNTD-D-20-00543

Another Dengue Fever Outbreak in Eastern Ethiopia – An Emerging Public Health Threat”

A detailed response to each comment is listed below. All changes in response to the reviewer’s comments have been incorporated into the manuscript text.

**Major points**

**All my major comments were addressed except the one on sequencing, and although I understand the reason raised by the authors (no sequencing facilities or capacity in the country), I think this should not prevent a researcher from finding a solution, especially with many commercial companies available at affordable cost in Europe.**

We would like to thank the reviewer for this comment. We agree with the reviewer’s suggestion regarding the importance of sequencing positive samples as well as with the affordable availability of commercial options. We regret to report, however, that since the initial revision of this manuscript was prepared, unforeseen circumstances have resulted in the removal of these specimens from storage. Due to the prioritization of the COVID-19 crisis and the resulting storage limitations resulting from widespread testing, the specimens described in this study were removed and replaced with COVID-19 samples. We apologize for this unexpected and unavoidable development.

As stated previously, this lack of sequencing is admittedly a limitation of our study and makes portions of our laboratory results observational and correlative when it comes to their relationship to past Dengue outbreaks. However, given the similar serotype makeup within the country and Somali Region, as identified by Ahmed et al. 2016, we feel that it is important to share our significant, albeit serologically-limited, findings. The timing, location, and risk factors associated with this outbreak add valuable information to the slowly growing database of Dengue in Ethiopia and can provide valuable insight toward future surveillance and prevention efforts. This point has been added to the discussion section of the revised manuscript in lines 485-489.

**Minor points (New)**

**I kindly advise the authors to consider reading and taking into consideration a recently published systematic review on dengue prevalence in Africa by Simo Nemg et al. 2019 in their introduction or discussion**.

Thank you for the suggestion. This review provided substantial enhancement to the manuscript and has been addressed in lines 116-124, 506-507, 508-509.

**Lines 150-152: I think this sentence sounds like a result, so please could you consider moving it to the appropriate section?**

This is an excellent point, and we appreciate the comment. This sentence has been relocated to the results section and now shown in lines 297-299.

**242-250: This paragraph of the method is a kind of results/discussion, so I kindly advise the authors to put it in the appropriate section.**

This is an excellent point, and we appreciate the comment. This sentence has been relocated to the discussion section and now shown in lines 405 – 409.

**Line 299: Please correct this sentence “Five cases of these cases presented…” as follow: “Five of the cases presented…”**

Thank you for pointing this out. The necessary correction has been made and is shown in line

302.

**My Previous (minor) comment:**

**Abstract: A total of 101 total cases… Please remove one total.**

**- Response of authors: We appreciate the reviewer’s comment, yet respectfully disagree with their sentiments. We decided to utilize the entirety of our study sample and to not remove 1 case. We agree 100 is a much more navigable and simplistic sample size to quickly understand, but felt the data is better served by including the entire sample.**

**My new comment:**

**The authors did not understand my comment here. I asked the authors to rephrase the sentence as follow: “A total of 101 cases…” without writing “total” twice.**

We apologize for the misunderstanding. The proper correction has now been made and is shown in line 45
